# The curious case of dermal fibroblasts: cell identity loss may be a mechanism underlying cardiovascular aging

**DOI:** 10.1093/cvr/cvz012

**Published:** 2019-02-20

**Authors:** Ileana Badi

**Affiliations:** Division of Cardiovascular Medicine, Radcliffe Department of Medicine, University of Oxford, John Radcliffe Hospital, Level 6 West Wing, Headley Way, Oxford, UK

**Commentary on ‘Identity noise and adipogenic traits characterize dermal fibroblast aging’, by Salzer *et al*., *Cell*, 2018.^3^**

The greater longevity achieved by our society has brought the current research to give a particular interest on understanding the mechanisms underlying ageing, as age is the major risk factor for many pathologies, including cancer, neurodegeneration, and cardiovascular disease.^1^ Ageing can be defined in general terms as a time-dependent structural and functional decline and the deterioration of the skin is one of the most apparent signs of it. The dermis, that is one of the three layers constituting the skin, is populated by fibroblasts that synthesize and secrete collagens and other matrix proteins that maintain the skin architecture and confer elasticity, resistance and strength to the tissue.^2^ During ageing the dermis is characterized by loss of cellularity and extracellular matrix (ECM) remodelling.^2^^,^^3^ Even though dermal fibroblasts have been widely used as an *in vitro* model of cellular ageing for decades, the molecular mechanisms leading to these cells ageing *in vivo* are poorly understood.^2^

Dermal fibroblasts derive from mesenchymal progenitors and can be distinguished in the newborn dermis in four types: the papillary fibroblasts, which are restricted in the upper dermis, the reticular fibroblasts that are spread throughout the ECM dense lower dermis and two Sca1^+^ pro-adipogenic types, which are located in the lower reticular dermis.^4^ However, in adulthood the cell surface epitopes that discriminate among these types of fibroblasts are lost and this compromises the possibility to investigate whether the adult dermis still contains these lineages.^4^ Salzer *et al.*, in a very recent issue of cell, addressed these gaps in our knowledge by means of bulk- and single-cell transcriptomic analyses and long-term lineage tracing. They found that during ageing dermal fibroblasts have a less well-defined identity, as the transcriptome features that define clusters in young cells become blurry with age, and paradoxically old fibroblasts acquire adipogenic traits reminiscent of newborn pro-adipogenic fibroblasts.^3^

The intrinsic rate of skin ageing in every organism can be hugely affected by extrinsic factors, such as exposure to ultraviolet light; since dermal fibroblasts are long-lived cells that continuously accumulate damage, they are a preferred model to study extrinsic ageing at the cellular level.^2^ However, although many studies display the beneficial effects of calorie restriction (CR) without malnutrition on longevity and age-related diseases, such as obesity, diabetes mellitus, cardiovascular disease, and cancer, the consequences of dietary interventions on dermal fibroblasts during ageing had been unexplored so far.^5^^,^^6^ Hence, Salzer *et al.*^3^ evaluated whether they could modulate the aged fibroblasts phenotype by feeding mice with a CR diet or a high fat diet (HFD). Interestingly, they found that CR could prevent the loss of papillary characteristics and the gain of adipogenic traits in old fibroblasts and that the transcriptome of adult dermal fibroblasts isolated from HFD-fed mice positively correlated with the one of old fibroblasts; these results indicate that CR and HFD could respectively delay and accelerate the ageing processes of these cells.^3^ Hence, they suggested loss of cell identity as a mechanism underlying cellular ageing and dietary intervention as a possible therapeutic strategy to slow down skin ageing. 

This study that employs state of the art techniques to decipher fibroblast ageing *in vivo* has surely a high impact in the poorly understood field of skin dermis ageing; but, I believe that it also has a broader relevance comprising the cardiovascular field. For instance, if loss of cell identity is a possible mechanism underlying ageing, this could also explain what occurs in the aged heart and vessels, as cardiac fibroblasts and vascular smooth muscle cells (VSMC), which similarly to dermal fibroblasts play crucial structural and functional roles in their tissues, are more prone to phenotypic shifts that lead to age-related dysfunctions, such as cardiac fibrosis and vascular calcification, respectively.^7–9^ Interestingly, both myofibroblasts and VSMC transdifferentiating towards an osteochondrogenic lineage display altered expression of ECM-related genes and increased expression of pro-inflammatory factors^9–12^; similarly, Salzer *et al.*^3^ observed that old fibroblasts, along with acquiring the newly identified feature of cell identity loss, exhibited these two typical characteristics of aged cells, as well as they up-regulated the expression of genes involved in adipogenesis. Although VSMC grown in apidogenic media develop adipocyte markers, it still has to be determined whether these cells, as well as cardiac fibroblasts, are able to acquire these adipogenic traits during ageing and, if so, it would be interesting to evaluate which are the consequences of this shift on the cardiovascular system.^9^

Intriguingly, the same cutting-edge approach of combining single-cell RNA sequencing and lineage tracing has been employed to unveil similar mechanisms in cardiovascular diseases. Indeed, Kretzschmar *et al.*^13^ in a very recent study observed that a subpopulation of activated fibroblasts acquires a neonatal-like gene expression profile in response to ischaemic injury; it is thus tempting to speculate that this cellular attempt of rejuvenation towards a neonatal-like state may be an adaptive mechanism that at least fibroblasts could adopt to respond to different insults, such as cardiac damage and skin ageing. Moreover, Dobnikar *et al.* investigated the transcriptional signatures of VSMC in healthy and atherosclerotic vessels and found a subpopulation of lineage-traced VSMC positive for the progenitor cell marker Sca1 that they suggested to be involved in the vessel response to injury; they also observed Sca1 up-regulation in VSMC exposed to stimuli that are known to induce the phenotypic switching of these cells.^14^ However, whether the loss of identity that may enable cells to acquire a stem/progenitor-like phenotype, possibly as an adaptive response to the age-related stem cell exhaustion, occurs in cardiac fibroblasts or VSMC during ageing still remains to be elucidated. 

Hence, the future research that aims to decipher the molecular and cellular mechanisms controlling cardiovascular ageing and age-related diseases should reckon with the findings described by Salzer *et al.*, that may be even extended to other organs and tissues.

**Conflict of interest:** none declared.

## Funding

This work was supported by the British Heart Foundation (FS/16/15/32047).

## Figures and Tables

**Figure:**
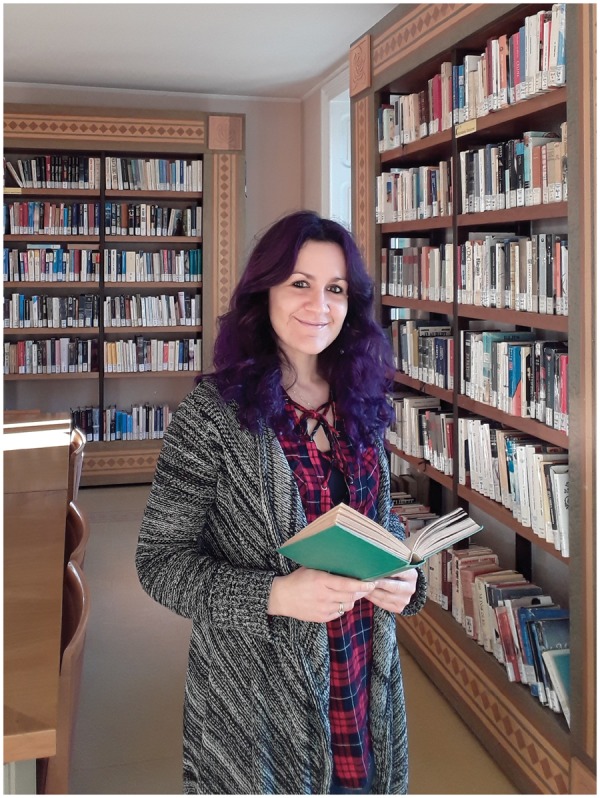


## Author

**Biography:** Dr Ileana Badi obtained her Ph.D. in 2008 at the University of Insubria (Italy), where she studied the role of the transcription factor Ankrd1 in the pathogenesis of the rare congenital heart disease Total Anomalous Pulmonary Venous Return (TAPVR). To deepen her knowledge on the transcriptional regulation during cardiac development she undertook post-doctoral research under the supervision of Professor Vincent M. Christoffels at the University of Amsterdam. She then moved to the Milanese Institute ‘Centro Cardiologico Monzino’ to focus on the post-transcriptional regulation in vascular biology, ageing and disease, as she mainly investigated the role of miR-34a in vascular ‘inflammaging’ and calcification. To pursue these more translational studies she had been awarded by the Italian Foundation ‘Fondazione Umberto Veronesi’ with its prestigious annual Post-doctoral Fellowship for four consecutive years (2015–2018). In June 2018, she joined ‘The Oxford Translational Cardiovascular Research Group’ at the University of Oxford to study how the cross-talk between adipose tissue and the cardiovascular system can affect cardiovascular disease and to lead the basic science laboratory of Professor Charalambos Antoniades. Dr Badi became a member of the Working Group on Atherosclerosis and Vascular Biology of the European Society of Cardiology (ESC) in 2015 and a member of the ESC Scientists of Tomorrow in 2018.
